# Tamsulosin 0.8 mg daily dose in management of BPH patients with failed tamsulosin 0.4 mg monotherapy and unfit for surgical intervention

**DOI:** 10.1007/s00345-024-05050-w

**Published:** 2024-06-01

**Authors:** Mohamed Mahmoud Dogha, Hossam Shaker, Assem Abdelazeez, Ahmed Abd-El Latif, Mahmoud S. ElAdawy

**Affiliations:** 1 Department of Urology, Fayoum University, Faiyum, Egypt; 2 Department of Urology, Beni-Suef University, Beni Suef, Egypt

**Keywords:** BPH, Unfit, Tamsulosin, Double dose

## Abstract

**Aim:**

This study aims to evaluate the effectiveness and safety of administering double-dose tamsulosin (0.8 mg) for treating patients with benign prostatic hyperplasia (BPH) who have not responded to the standard single dose of tamsulosin (0.4 mg) and are deemed unsuitable for transurethral resection (TUR) intervention.

**Materials and methods:**

Between November 2022 and July 2023, we prospectively analyzed 111 patients who were experiencing severe BPH symptoms. These patients received a double dose of tamsulosin for one month. We collected baseline characteristics such as age, body mass index, and underlying medical conditions. Various parameters including the International Prostate Symptom Score (IPSS), prostate-specific antigen (PSA) levels, prostate volume, peak urinary flow rate (Qmax), voided volume, and post-void residual volume were evaluated before and after treatment.

**Results:**

All 111 patients completed the study. The mean age, PSA level, and prostate volume were 63.12 ± 4.83 years, 3.42 ± 0.93 ng/ml, and 50.37 ± 19.23 ml, respectively. Of these patients, 93 showed improvement in Qmax, post-void residual volume, and IPSS score (*p*-value = 0.001). The total IPSS score and total Qmax improved from 24.03 ± 2.49 and 7.72 ± 1.64 ml/sec to 16.41 ± 3.84 and 12.08 ± 2.37 ml/sec, respectively.

**Conclusion:**

Double-dose 0.8mg tamsulosin as an alpha-blocker therapy appears to be a viable temporary management option for BPH patients who have not responded to the standard single dose 0.4mg tamsulosin and are not suitable candidates for TUR intervention.

## Introduction

Lower urinary tract symptoms (LUTS) associated with benign prostatic hyperplasia (BPH) are prevalent among older men, affecting around 25% of those aged 40 and above according to community-based surveys [1]. Clinical BPH can disrupt daily activities and diminish health-related quality of life, particularly concerning urinary symptoms. Treatment options encompass watchful waiting, lifestyle adjustments, herbal remedies, prescription drugs, and surgical procedures. Medical management stands as the primary therapeutic approach for many symptomatic BPH patients [2]. The overarching objective of medical management is to alleviate short-term symptoms, mitigate treatment side effects, and ultimately prevent complications while maintaining quality of life. Currently, alpha-1-adrenergic receptor antagonists (alpha-blockers) and 5-alpha-reductase inhibitors (5-ARIs) represent the most efficacious medical therapies for BPH [3]. Alpha-blockers alleviate symptoms and enhance urinary flow rate by relaxing prostatic and bladder neck smooth muscles through the blockade of sympathetic activity. Alpha-blockers, including doxazosin, terazosin, prazosin, alfuzosin, and tamsulosin, are the most commonly prescribed medications [4]. Tamsulosin, distinguished by its high affinity for alpha-1a-adrenergic receptors, predominantly mediates prostate and bladder smooth muscle tone, thus improving the dynamic component of bladder outlet obstruction (BOO) and promptly relieving LUTS. Unlike other non-selective alpha-blockers like doxazosin, alfuzosin, and terazosin, tamsulosin is well-tolerated due to its prostate selectivity [5].

All 1-adrenoceptor antagonists seem to have similar efficacy in improving symptoms and flow. The difference between ·1-adrenoceptor antagonists is related to their side effect profile. Alfuzosin and tamsulosin appear to be better tolerated than doxazosin, terazosin and prazosin [6].

Due to its prostate selectivity, tamsulosin may ameliorate urinary symptoms and flow with fewer adverse effects. Numerous studies have demonstrated the efficacy and tolerability of tamsulosin at doses ranging from 0.2 to 0.8 mg once daily in symptomatic BPH patients. Several trials, including those conducted in Japan, China, and Korea, have investigated tamsulosin's efficacy at 0.2 mg [7–9].

In our study, we explored the effectiveness and safety of double-dose tamsulosin in treating BPH patients who did not respond to the standard single dose and were not suitable candidates for transurethral resection intervention.

## Patients and methods

Our prospective study, conducted from November 2022 to July 2023 across various Urology clinics in Cairo and Fayoum, Egypt, focused on patients over 50 years old who presented with severe lower urinary tract symptoms (LUTS) related to benign prostatic hyperplasia (BPH) despite being on tamsulosin single dose 0.4mg. Initially, 150 patients were enrolled, but only 111 completed both visits, resulting in 39 dropouts. Inclusion criteria comprised a minimum of 3 months of previous tamsulosin monotherapy, an International Prostate Symptom Score (IPSS) exceeding 19, a peak urinary flow rate (Qmax) below 10, and being unfit for surgery due to medical conditions or refusal. Patients on treatments other than tamsulosin were excluded. Comprehensive interviews, examinations, and investigations including IPSS, Quality of Life (QoL), prostate volume, Qmax, and abdominal/pelvic ultrasound were conducted at presentation and at the 1-month visit. A responder group was identified based on an IPSS score improvement of more than 3, with ethical committee approval obtained.

### Statistical methods

Data were analyzed using SPSS version 28, summarized using mean and standard deviation, and compared using paired t-tests (*p* < 0.05 considered significant) [14].

## Result

**Fig. 1:**
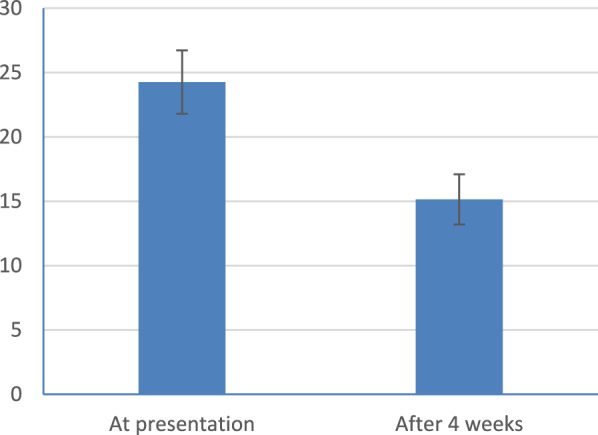
Symp.score IPSS at presentation and after 4weeks (responder)

**Fig. 2:**
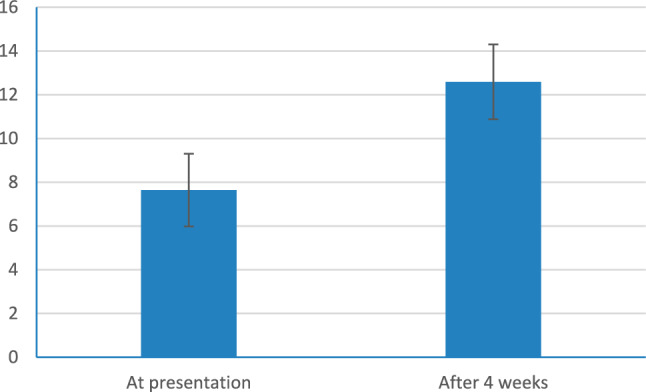
Uroflow Q max ml/sec at presentation and after 4 weeks

**Fig. 3:**
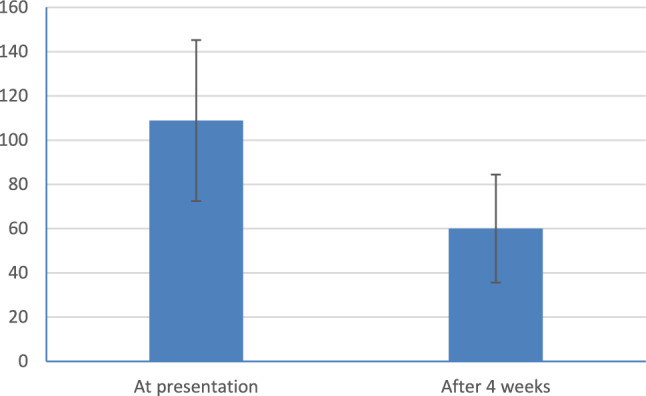
Residual urine at presentation and after 4 weeks

A total of 150 patients were enrolled at the initial visit. but only 111 patients completed the two visits, thus 39 patients were dropped out. Total Qmax improved from 7.72 ± 1.64 ml/s to 12.08 ± 2.37 ml/s (*p* = 0.001) and Total IPSS Sympom Score improved from 24.03 ± 2.49 to 16.41 ± 3.84 (*p* = 0.001). The IPSS scores of 93 patients had increased by more than 3 and they were assigned to the responder group while the remaining 18 patients, whose IPSS scores had not increased by more than 3, were assigned to the non-responder group.The general characteristics of the patients in the two groups were similar with respect to age and PSA. Mean age was 63.12 ± 4.8 years and mean PSA 3.42 ± 0.93 ng/ml. Prostate volume was significantly larger in the responder group (51.68 ± 19.58 ml vs, 43.61 ± 16.14 ml, *p* = 0.001).also in the responder group, IPSS score and maximal urine flow rate improved from 24.26 ± 2.46 and 7.64 ± 1.66 ml/sec to 15.14 ± 1.95 and 12.59 ± 1.71 ml/sec, respectively (*p* value < 0.001) Table 1, Figs. 1,2 and PVR urine improved from 108.84 ± 36.38 ml to 60.02 ± 24.40 (*p* value < 0.001) Fig. 3. In the non-responder group, IPSS score and maximal urine flow rate changed from 22.83 ± 2.38 and 8.14 ± 1.49 ml/sec to 23.00 ± 4.49 and 9.47 ± 3.45 ml/sec, respectively (*p* value < 0.815) and PVR urine changed from 110.78 ± 15.38 ml to 104.14 ± 42.88 (*p* value < 0.524). The mean QoL of the responder group at baseline and 4 weeks was 4.69 ± 0.78 and 4.10 ± 0.99 with *p* value < 0.001 respectively Table 1. On the second visit, out of the 111 patients included in our study, 6 patients (5.4%) stated that they experienced dizziness, 4 (3.6%) patients complained from decreased semen or abnormal ejaculation that they did not have before. 7 patients (6.3%) complained of mild non specific symptoms as (Headache, Back pain, Diarrohea, nausea, body aches, blurred vision).

**Table 1:** Patients characteristics, IPSSscore,Q max, residual urine and QoL at presentation and 4 weeks

	Total	Responder (n = 93)	Non-responder (n = 18)	*p*-value
Age, years	63.12 ± 4.83	63.04 ± 4.67	63.50 ± 5.70	0.715
PSA, ng/ml	3.42 ± 0.93	3.42 ± 0.93	3.43 ± 0.96	0.946
IPSS total score				
Baseline	24.03 ± 2.49	24.26 ± 2.46	22.83 ± 2.38	
At 4 weeks	16.41 ± 3.84	15.14 ± 1.95	23.00 ± 4.49	
p-value	< 0.001	< 0.001	0.815	
Qmax (ml/sec)				
Baseline	7.72 ± 1.64	7.64 ± 1.66	8.14 ± 1.49	
At 4 weeks	12.08 ± 2.37	12.59 ± 1.71	9.47 ± 3.45	
p-value	< 0.001	< 0.001	0.097	
PVR (ml)				
Baseline		108.84 ± 36.38	110.78 ± 15.38	
At 4 weeks		60.02 ± 24.40	104.14 ± 42.88	
p-value		< 0.001	0.524	
QoL				
Baseline	4.68 ± 0.77	4.69 ± 0.78	4.61 ± 0.69	
At 4 weeks	4.08 ± 0.97	4.10 ± 0.99	3.89 ± 1.02	
p-value	< 0.001	< 0.001	0.010	

*t-test

**Paired Samples Correlations

## Discussion

Benign prostatic hyperplasia (BPH) is a progressive condition characterized by worsening symptoms over time and, in some cases, the need for surgery. Given the associated risks of surgery and prolonged catheterization, our study explored the use of double-dose tamsulosin as a temporary solution for unfit patients.

Numerous studies with doses of 0.2, 0.4, 0.6, and 0.8 mg have been done to determine the efficacy and safety of tamsulosin for the medical treatment of BPH. The initial study by Abrams and associates was undertaken to help to establish the efficacy and safety of tamsulosin, as well as to determine the optimum dosage for treatment [10]. Patients were randomly assigned to placebo, 0.2, 0.4, or 0.6 mg of tamsulosin once daily for 1 month. The greatest reduction in symptoms occurred in those taking either 0.4 or 0.6 mg compared with 0.2 mg and placebo [10]. Also The two highest doses provided the greatest improvement in uroflow compared with placebo. There were no dose-related changes in vital signs or laboratory variables [10].

Although effect of various doses of tamsulosin have been investigated in BPH patients in many articles over the years. Yet in our study we have studied the effect of increased dose in a unique populaton of BPH patients,we targted BPH patients with severe symptom profile whom were indicated to do surgery, but they were unfit to do it.

The primary objective of the present study was to evaluate the efficacy of double dose tamsulosin compared with single dose tamsulosin for the management of prostatic patients with moderate to severe symptoms and unfit for surgery. Our results as regard to IPSS and Q-max flowmetry and PVR urine were improved with p value < 0.001.

*Yasuhiko Hirose *et al. showed no significant alteration in IPSS total score or QOL score with the increased dose of tamsulosin, but Qmax improved from 10.1 ± 5.5 ml/s to 12.1 ± 6.5 ml/s (*p* = 0.013), and residual urine volume improved from 37.6 ± 26.4 ml to 22.2 ± 24.3 ml (*p* = 0.012) [11].

*Chung *et al. *2011* reported that There were significant differences in IPSS, QoL, and Qmax at week 8 in both groups. There were significant differences in improvement in IPSS, QoL, Qmax, and postvoid residual urine volume from baseline to week 16 in both groups. There were no significant differences in efficacy or tolerability between the groups at week 16. At week 16, the mean change in the IPSS from baseline was − 7.0 ± 6.2 and − 5.1 ± 5.2 in the 0.2 and 0.4 mg groups, respectively [12].

*Park *et al. *2012,*reported that after tamsulosin 0.4 mg, there was a significant improvement in maximal urine flow rate in all patients. The mean total IPSS score of the 31 patients (31/60, 51.7%) who responded to tamsulosin 0.4 mg increased by more than 3 and PVR, improved significantly. But total IPSS score did not improve in about half of the patients (29/60, 48.3%) after tamsulosin dose escalation [13].

We reported that Prostate volume was significantly larger in the responder group (51.68 ± 19.58 ml vs 43.61 ± 16.14>ml, *p* = 0.001) as also *Park *et al. *2012* demonstrated Prostate volume was significantly larger in the responder group (33.0 ± 8.7 ml vs. 28.7 ± 6.0 ml, *p* = 0.032) and this may be explained by more saturation to receptors in prostatic tissue occurred with double dose tamsulin 0.8 mg.

For drug related adverse events the results were statistically insignificant between other studies. In our study adverse events were low and comparable across studies which are made with selective α1- blockers.

## Conclusion

Double the dose of tamsulosin 0.8 mg is worth trying to improve symptoms of BPH for unfit patients for surgical intervention with good safety and efficacy.
